# Contemporary myths on boredom

**DOI:** 10.3389/fsoc.2023.1183875

**Published:** 2023-06-07

**Authors:** Josefa Ros Velasco

**Affiliations:** Department of Philosophy and Society, Faculty of Philosophy, Complutense University of Madrid, Madrid, Spain

**Keywords:** boredom, contemporaneity, creativity, culture, modernity, myths & facts, pathology, popular narrative

## Abstract

*We don't know almost nothing about boredom*. Even though the experience of boredom has been part of our daily life for centuries, we are far from being clear about what its suffering consists of, what its main causes and consequences are, or how we can satisfactorily escape it. This is one of *the most repeated myths* about boredom among some boredom scholars; one from which many others derive, causing unnecessary confusion about a phenomenon around which there exists, in fact, a whole corpus of scientific knowledge. Most of them are harmless, simple narratives from our popular culture; others, however, have the power to condition the way in which we perceive reality, to the point of becoming stigmatizing. Breaking with some of our most ingrained beliefs about boredom is not an easy task, although it is necessary to understand the true nature of this state. In my essay, I will try to disprove some of the contemporary myths that circulate about the experience of boredom. Starting with the first myth, I will explore the scope of other related myths such as those that say that *the study of boredom is in its infancy*, that *boredom has not been given the attention it deserves*, that *the experience of boredom is born in modern societies*, that *boredom is an exclusively human condition*, that *boredom only happens in leisure time*, that *being bored is the same as doing nothing*, that *it is desirable to have moments of boredom*, that *boredom helps our brain to rest*, that *boredom makes us more creative*, and that *those who get bored is because they want to* or, what is worse, that *only fools get bored*.

*We don't know almost nothing about boredom*. Even though the experience of boredom has been part of our daily life for centuries, we are far from being clear about what its suffering consists of, what its main causes and consequences are, or how we can satisfactorily escape it. This is one of *the most repeated myths* about boredom among some boredom scholars; one from which many others derive that, at present, cause unnecessary confusion about a phenomenon around which there exists, in fact, a whole corpus of scientific knowledge. We are bombarded each day—especially by the press—with false mantras about boredom that end up occupying a place in the collective imagination. Most of them are harmless, simple narratives from our popular culture; others, however, have the power to condition the way in which we perceive reality, to the point of becoming stigmatizing. Breaking with some of our most ingrained beliefs about boredom is not an easy task, although it is necessary to understand the true nature of this state. In tribute to what has been achieved to date by experts in Boredom Studies, all that remains is an exercise in demystifying the subject of boredom.

I shall begin by returning to the first announced myth. On numerous occasions, I have heard colleagues who work on boredom, in encounters of all kinds, say that we know almost nothing about this common state. Our alleged unawareness of what boredom is cannot be justified in any way whatsoever; not just because we all suffer from this in person, with different intensities and duration, but also because it is unfair to those thinkers who, since ancient times, have devoted their efforts to defining such a peculiar state. Starting with classical authors like Horace, Lucretius and Seneca, through to contemporary researchers like Elpidorou, Eastwood and Van Tilburg, boredom has been described as a state of unrest caused by an imbalance between what we expect from the context we find ourselves in and what we truly feel we gain from it; a turmoil that leads us, firstly, to re-evaluate our present situation and then to search for the change that re-establishes our sense of balance with the environment (Ros Velasco, 2022a).

During the Roman empire, these mentioned philosophers wrote about the experience of boredom in its simplest form (*fastidium, satietas, desidia*) and in its most complex state (*taedium vitae*), defining it as heaviness, lentitude or satiety, due to an excess of idleness, leading to mental disorders, irritation and apathy (Seneca, 2004, 2009, 2014; Lucretius, 2008; Horace, 2011). In the Middle Ages, boredom, known as *acedia*, meant the alienation of the soul for St. Augustine of Hippo (2006, 2007, 2008) and Ponticus (2006) described it as despondency and sadness compared with a fulfilled life; Cassian (1999, 2015) used the terms weariness, disgust and anxiety of the heart to characterize it; John Chrysostom (PG 47.425-426) indicated that it was a destructive spiritual condition generated by a lack of perceived sense and desperation; St. Jerome (PL 22.1081-82) assimilated it with melancholy caused by loneliness; Hugh of Saint Victor (PL 176.525-526) mentioned that it was a disturbance of the mind caused by anxiety and St. Thomas Aquinas (1993, 1995, 2017) defined it in terms of a lack of joy and appetite for and interest in things.

In the Renaissance, writers like Petrarch (1991, 1992, 1999, 2016) and Dante (2014) alluded in their works to the lack of virtue and the inability to find hope or desire that defines boredom, that old sadness that torments both body and soul. Others, like St. Ignatius of Loyola (1992, 2014) and St. John of the Cross (2005) considered it to be a darkness of the soul or restlessness without hope, idleness or tepidity, bad mood or a physical malaise that results in displeasure, while Ficino (2019) spoke of a melancholic boredom leading to genius which, in excess, afflicts the soul with permanent tedium.

Great thinkers form the Enlightenment like Voltaire (1838, 2003, 2017) and Kant (2006; see also Ros Velasco, 2021a) referred to boredom as a consequence of excess free time and a lack of work, a concept which the German romantic writer von Kleist (2003) would also later agree with. Along the same line, some of his contemporaries analyzed our social structure to approach boredom. Heine (2005, 2006, 2016) considered it to be a symptom of the inflexibility and oppression induced by society, while Tieck (1828, 1853, 1854) understood boredom to be a stagnation of the soul or a murky defect of life, a cultural exhaustion resulting from a distressing and exhausting psychic misery. Additionally, Büchner (1998) declared that boredom—the *Langeweile*—was an individual inability to make sense of our surroundings.

The French intellectuals of the 19th Century, for their part, made an in-depth analysis of the experience of boredom in a host of philosophical literary works. For the Viscount of Chateaubriand (1952, 2014), boredom meant suffering from a serious paralysis of one's will. de Senancour (2010) expressed boredom as an opposition between what one imagines and what one experiences, a state in which one does not know what to wish for precisely because these wishes are disproportionate, and the imagination has promised too much. This is similar to what the authors Staël-Holstein (1835, 1995, 1998, 2000, 2010) and Sand (2022) advocated. A curious case is that of Flaubert (2003), the creator of one of the characters most afflicted by boredom of all time—Bovary. The novelist explicitly differentiated, for the first time in history, between two types of boredom (Flaubert, 1980): the banal tedium due to an excess of leisure time and the modern tedium of an existential nature—what we now know as simple or passing boredom, in contrast to complex or profound boredom. Baudelaire (2017) filled dozens of pages about this latter concept, describing it as desperation or the angst of hopelessness because everything one aspires to is unrealistic; the absence of desire is also what Tolstoy (2017) spoke about. Furthermore, other philosophers like Kierkegaard (2013) ascribed to the thought on this form of profound boredom that leads to angst.

Beyond philosophy and literature, many physiologists were interested in the state of boredom in the French context at the end of the 18^th^ and 19^th^ Centuries. Hallé and Thillaye (1819) suggested that this was a case of a painful torment that brought with it considerable disarray; Esquirol (1821) appreciated it as a form of hatred of life; de Sauvages (1771) as a disease stemming from tiredness with existence; Littré (1863) as a vacuum of the soul deprived of an interest in things, while de Boismont (1850) referred to it as a moral disease of modern society. His understanding of boredom fully coincided with that of other philosophers with a sociological bias—the treatment of boredom from a purely sociological perspective has not begun to gain visibility until recently, thanks to works such as those by Conrad (1997), Gardiner and Haladyn (2016), or Ohlmeier et al. (2020), for example—like Simmel (1950, 1983), Kracauer (1995), Marx (2009), Lefebvre (2012, 2022), and Adorno and Horkheimer (2016), to mention but a few, for whom tedium was just a symptom of capitalism and its worst creatures—among them, the culture of mass entertainment.

The 20^th^ Century was a period of greater scientific production regarding this phenomenon. Until that time, the only work exclusively given over to the analysis of boredom was that of de Boismont (1850), *De l'ennui, taedium vitae*. But it was soon added to this the work *L'ennui. Étude psychologique*, by Tardieu (1913). This French psychologist spoke about boredom as turmoil and neglect, as a nostalgia for the unattainable and skepticism of having lived too much; a condition that could even become chronic. When Münsterberg (1913) turned boredom into a scientific research matter based on his study of performance in factories, showing it as a sentiment of monotony which depended on one's individual disposition, many other researchers in the field of mental health began to pay attention to boredom with unusual generosity. Lipps (1909), albeit a philosopher, defined it through a psychodynamic theory as a conflict between the internal need for an intense mental activity and a lack of stimulation or the subject's inability to find stimulation. Fenichel (1951), a disciple of Freudian psychoanalysis, observed that tedium was a state of tension characterized by the co-existence of a need for activity and the dissatisfaction with future stimulation.

In the mid-20^th^ Century, researchers from the field of psychology, coming from a range of very different movements such as those based on the arousal theory or cognitive theory, among others, focused on discovering the intricacies of boredom more in-depth. Listing their conclusions here would exceed the space available to me and the aim of this essay, but an exhaustive outline can be found in Chapter 6 of my book *La enfermedad del aburrimiento* (*The Disease of Boredom*) (Ros Velasco, 2022a). Among the most cutting-edge definitions is that of Greenson (1951, 1953), regarding apathetic and agitated boredom; that of Warren (1934), who explained that tedium was an unpleasant condition of foggy attention, resulting from the automation of an activity and the difficulty in finding change; that of O'Hanlon (1981), according to whom boredom was a psychophysiological state produced by lengthy exposure to monotonous stimulation; that of Csíkszentmihályi (1975, 1998, 2000), creator of the well-known flow theory, who treated boredom as a psychological state of dissatisfaction caused by a decline in neurological stimulation as a result of the experience of uninteresting or repetitive situations; that of Mikulas and Vodanovich (1993), who pointed to a state of low stimulation and dissatisfaction attributable to an unsuitable environment; that of Sundberg (1994), who distinguished between passing boredom (state-boredom) and boredom as an individual trait or a recurring disposition (boredom proneness), or that of Campbell (1996), for whom tedium was a sentiment of displeasure due to a need for greater activity or to a lack of significant stimulation or an inability to perceive the stimulation.

At present, some of the “popes” of boredom continue to work tirelessly to complete the profile of an unequivocal definition of this state. Eastwood et al. (2012) tell us that boredom is a state of aversion that primarily occurs when we are unable to satisfactorily commit to the internal or external information required to take part in an activity, when we are aware that we cannot maintain our attention to gratifying activities because they demand a high degree of mental effort and when we attribute the cause of our state to the environment. Their argument was fine-tuned later on by the researchers Westgate and Wilson (2018) in what is known as the MAC Model. Van Tilburg and Igou (2011, 2017, 2019) and Van Tilburg et al. (2022) stressed the role played by the lack of meaning perceived in a situation or an activity in preventing our commitment to it, which is what leads to boredom. In the realm of neuropsychology, Danckert (2018, 2019) proposed that boredom is an omnipresent human experience that can be described as the inability to interact with one's environment despite being motivated to do so. Lastly, the philosopher Elpidorou (2014, 2017) examined the functional component of boredom, presenting it as a state that warns us of situations that are not valuable to us and should thus be abandoned.

After this long journey—which can be looked at in-depth in my book (Ros Velasco, 2022a)—the reader will agree that it is hard to maintain the claim that we know almost nothing at present about boredom. With different nuances, all these thinkers have contributed to us all having an idea, even if minimal, about what this state constitutes and implies, which can be expressed as follows: boredom is a state of malaise that we suffer from when the environment in which we find ourselves immersed or the activity we try to engage in does not stimulate us in line with our initial expectations, resulting in the painful experience of meaninglessness. We all suffer from this, more or less frequently, at different times and in different places, depending on both exogenous factors that stem from the possibilities of the context, and endogenous related to one's own personality and expectations. The person who is bored feels that their relationship with the present reality is damaged and they should do whatever within their grasp to return to an optimum state of stimulation, which translates into the sense of wellbeing yearned for (Ros Velasco, 2022a).

This description is applicable to any experience of boredom in which what determines its ultimate expression is the interaction between such variables as intensity (superficial or profound), durability (passing or chronic), and the agent of the experience (individual or collective) in this relationship established between the person (or people) bored and the specific context (Ros Velasco, 2022a). These are some of the variables quoted by Flaubert (1980) when classifying boredom in the categories of banal and existential tedium (Greenson, 1951, 1953, however, was guided by the type of response elicited to address the boredom to classify it as agitated or apathetic). Heidegger (1995) also tested out his own classification of boredom—being bored with something (*das Gelangweiltwerden von etwas*), boring oneself with something (*das Sichlangweiligen bei etwas*), and profound boredom (*das Sichlangweiligen*); a distinction that I have serious discrepancies with (Ros Velasco, 2022a, see Chapter 7, in particular). I feel the classification by the poet Valéry (1951) is more complete, for whom boredom could be transient (*l'ennui passager*), due to weariness (*l'ennui par fatigue*) or with life (*l'ennui de vivre*). The classification by Valéry may be considered the predecessor of what I have proposed myself (Ros Velasco, 2022a), according to which, based on the variables mentioned, boredom may be situation-dependent and transient, situation-dependent and chronic, individual-dependent and chronic, and existential-profound, always taking into account the perspective of its functionality or dysfunctionality insofar as we are capable or not (for endogenous or exogenous reasons) of reacting to prevent suffering from it. Classification aside, the essence of boredom—even addressing it as a multifactor phenomenon—is always the same: this unrest as a result of a relationship with the present that has become obsolete and that we have to address. I believe we can all see a part of ourselves in this “simplification”; hence, we know something, if not a lot, about boredom.

I have taken the time to debunk this myth because, as I warned at the beginning, any subsequent ideas are a result of this. Without going further, the following myths are two typical responses to the question: why don't we know *almost nothing* about boredom? Of course, we don't know anything because *the study of boredom is still in its infancy* and because *boredom has not been paid the attention it deserves* (see e.g., O'Hanlon, 1981; Smith, 1981; Farmer and Sundberg, 1986; Damrad-Frye and Laird, 1989; Pediaditakis, 1991; Fisher, 1993; Leong and Schneller, 1993; Scitovsky, 1999; Watt and Vodanovich, 1999; Martin et al., 2006; Pekrun et al., 2010; Thompson, 2020). I am not surprised that those who are not engaged in researching this phenomenon think that boredom has not aroused our curiosity as a subject of scientific analysis until a relatively short time ago. What I do find surprising is that these claims are made by some “boredom scholars” (Ros Velasco, 2017).

Nearly 100 years have elapsed since Russell (1930) denounced, in *The Conquest of Happiness*, that the study of boredom was being ignored; 100 years in which this “study of boredom” has become an exceptional subject of interest. Aside from everything that the philosophers, theologians, authors and physiologists have said in centuries gone by—if we brought it all together, the calculation would amount, without exaggerating, to several thousand pages (Ros Velasco, 2017)—during the first half of the 20^th^ Century, more than ten works were published about boredom; while in the second half, the number of publications stretched into the hundreds (Ros Velasco, 2017). With the dawn of this new century, the number of studies about boredom easily exceeds anything written before. By way of example, in 2013 alone, a total of 119 papers were published (Ros Velasco, 2017).

However, this evidence does not prevent many researchers from justifying the need for their works based on these two myths (from the texts by O'Hanlon, 1981 at the end of the 20^th^ Century, to more recent ones, drafted by experts like Pekrun et al., 2010), although the tendency to identify a gap in the literature as a strategy to justify one's own work is not exclusive to this field of study. As well as myself, some colleagues like Goodstein (2020) and Finkielsztein (2021) call for this false belief to be banished once and for all—without much success, it should be said. It is true that the study of boredom has been very disperse in terms of disciplines (Ros Velasco, 2017). It is also true that its institutionalization and academization is only just beginning now (for example, with the launching of the International Society of Boredom Studies in 2021 and the opening of labs such as the Danckert Lab or the Boredom Lab). That is not reason enough to maintain that we have barely begun to research boredom or that in the past we have not shown an interest in the experience of boredom.

Such affirmations only make sense in very specific contexts; for example, when applied to the study of boredom in animals in the field of zoology (see e.g., Meagher, 2019), or the analysis of boredom in people with Alzheimer's in the field of psycho-gerontology (Ros Velasco, 2021b). Let's change the expression “the study of boredom is in its infancy” to “the study of boredom in X is in its infancy” and the expression “not enough attention has been paid to boredom” to “not enough attention has been paid to boredom in X”, if that were the case.

Another myth related to these—which has been debunked in what I have set out above—is that which states that *the experience of boredom has arisen in modern societies* (see e.g., Meyer Spacks, 1995; Svendsen, 2004; Goodstein, 2005). Boredom is not the exclusive jurisdiction of capitalist societies, originating in the modern world (Ros Velasco, 2022a,b)—although the causes of boredom, the characteristics of its experience, and its consequences are different in modern times compared to other historical periods insofar as they respond to its particularities. According to the description I have suggested, boredom is within the grasp of anyone—even for species other than ours, which debunks the myth that *boredom is an exclusively human condition* (see Wemelsfelder, 1985; Meagher and Mason, 2012; Svendsen, 2019a,b). Many people believe that hunter-gatherer societies did not suffer from boredom because their lives were very busy in an attempt to survive. They are wrong. Our predecessors invested a lot less time in survival than we employ nowadays (see Lee, 1979; Le Guin, 1989; Barnard, 2016; Sahlins, 2017). But even if this were the case, filling our time with numerous tasks does not imply an absence of boredom: these may be very unstimulating tasks. At any event, modern boredom is attributed to the birth of leisure time; time that other civilizations, like the Romans (see Ros Velasco, 2022a, particularly Chapter 1) also enjoyed.

It is easy to think that boredom is experienced more in modern times than in any other because we have more free time in which this state can appear. But, in the same way as we cannot suppose that we simply avoid getting bored by being busy, nor should it be supposed that the availability of free time means it is easier to suffer from boredom. Boredom can arise as easily in leisure time as in duty time. What distinguishes us from other times is that there are many more of us with the chance to write about our experiences, in whatever context. Of course, those that suffer from boredom because they do not know how to manage their free time are also those that have more time to reflect on their experiences, in this specific situation, for posterity. Those that are bored by endless days of work do not tell us this because they have no time to do it, but that does not mean that they are not bored—this is why the issue of boredom in the workplace is still as heavily scrutinized today as it was a century ago (Butler et al., 2011). This may give us the sensation that boredom is something modern as a result of an excess of free time, of the emergence of new lush societies as a consequence of the industrial revolution (Veblen, 1994), but, as I already said, having free time is not something exclusive to modern-day societies, nor is writing about it—although it is a period that facilitates this task—nor *does boredom exclusively take place in free time*; another myth that has been debunked, together with those popular sayings that exclaim that *only the rich get bored* because they have a lot of free time or that *boredom is the same as doing nothing*.

We get bored when we are obliged to do nothing, when what we would like is to be doing something that we have chosen to do ourselves, the same as we get bored when we have to do something by external demand that is not important or stimulating to us, when we would prefer doing whatever we want or doing nothing at all (Ros Velasco, 2022a). Not doing anything because that is what we have prescribed for ourselves—because we want to rest, disconnect or reconnect with our own thoughts—is not synonymous with boredom under any circumstance. This ties in to another popular myth: that of *wanting to have time to be bored*. Boredom is always painful because it is the result of dissatisfaction. No-one in their right mind yearns to feel pain, except those who believe that suffering is necessary to reach a high level of existence. What we want is to have free time (Blumenberg, 1986) to carry out activities that are freely chosen or to do nothing at all. But we do not expect that, at the end of the day, boredom will occupy this free time we have. Everyday expressions like “I plan to spend the weekend bored” or “if only I had time to get bored” stem from a confusion in the meaning we attribute to the word “boredom”. You only have to think about a time when you were really bored, reading a book you couldn't get in to or having to wait in a waiting room without the chance to escape, and relive this malaise you felt in order to see that you wouldn't want to repeat this situation or for your boredom to have lasted any longer. Tedium is not pleasurable, unless when we say “tedium”, what we really want to allude to is “rest”, which is totally the opposite. Maybe we should extend the definition of boredom to include this new broader view, whereby “boredom means enjoying doing nothing of your own volition”, although, for this meaning the Oxford English Dictionary has coined the term “to be in goblin mode”.

When we say we want to have time to get bored, in reference to the positives of having free time, it seems like boredom only occurs in this leisure time, when we have seen that boredom can occur both in our free time and in undertaking tasks—for example, carrying out a repetitive job. Even after debunking this myth, many people advocate the idea that, whenever boredom may occur, whether in free time or duty time, it is useful to experience it because *boredom makes us more creative and smarter* (for examples, just google the words “boredom” and “creativity”). This is my favorite myth because it is one of hope and resilience which makes it desirable to have time for tedium.

We endlessly hear repeated in the media that boredom is positive because it drives creativity, and hence it is desirable to have time to be bored and spend long periods of time doing nothing. I won't stress anymore that people can get bored anytime and whether you are doing something or nothing. Let's focus now on this issue of increased creativity (being smart is only a knock-on effect of this). When we get bored, we feel pain—that is clear—and, as people that flee from pain and pursue pleasure, we try to put a stop to what we find to be a nuisance by making use of the resources within our grasp—these are different depending on the context and on each person (Ros Velasco, 2022a). To express this another way, when exposed to a boring situation or activity, our levels of cortical stimulation drop, and we feel a malaise that forces us to design a strategy to recover our equilibrium enjoyed before this exposure. What we do to make the source of boredom go away is introduce a change into our environment. It is in this process that some people detect a component of creativity.

Escaping from boredom implies a moment of “creativity”, in the sense that we must bring into play something where previously there was nothing or where what there was didn't stimulate us. However, what we are told through this myth is that boredom will help make us more creative, in reference to imagination or invention, which is to suppose a great deal. Boredom is reactive—I prefer to use this word to avoid the confusion generated by the term “creative”—a driving force that boosts change but, firstly, this change does not necessarily imply the introduction of something completely new and original into the environment (Ros Velasco, 2023). In fact, usually, the design of this strategy to flee from boredom does not even take place consciously, but rather we resort to ways stored away in our subconscious that have proven to be successful in the past in disarming the source of boredom; we do this almost mechanically: if a film bores me, I change to a series or play a videogame or connect to social media or I call my friends to go out and have some beers or I read a book. Even when we become aware that we need to do something to prevent boredom and we start to think about how to do this, we end up resorting, time and time again, to the same common solutions. Obviously, we cannot generalize, not one way or the other.

The belief that boredom makes us more creative exudes great optimism. It not only seeks to convince us that something original can come out of our boredom, but that this creation stemming from the response to boredom will be useful. We forget that a large percentage of our reactions (conscious or subconscious) are dysfunctional or unhealthy (Ros Velasco, 2017, 2022a; Sommer et al., 2021). If tedium is going to make me more creative in the sense that it will arouse my curiosity in drugs, perhaps the supposed creativity that inspires me is not as welcome as we would like to think. The reactive nature of boredom is praiseworthy because it keeps us in motion, but not because it leads to geniality. Never in the history of the study of boredom has this state been observed from such a perspective, perhaps with the exception of the Renaissance, when it was believed, as Ficino stated, that boredom, in its most profound form, made us great (see also Ros Velasco, 2022a, especially Chapter 3). It is true that creative people are able to make better use of their boredom in that they respond to this with more original reactions (Ros Velasco, 2023), but this only means that boredom drives the creativity of creative people or the geniality of the genius, as furthermore also happens with the destructivity of destructive people (Ros Velasco, 2023). Pursuant to the foregoing, the answer is “no”; boredom does not necessarily make us more creative (not even children). We often overestimate our ability to respond creatively and functionally to boredom.

These slogans also send us the message that, on the other hand, it is negative to spend all our time busy doing things; a direct criticism of the society of hyperactivity. One of the main complaints of our century is that we do not leave room for boredom in the midst of the endless cascade of activities that infest all the hours of the day, all the days of the week that span all the months of the year (despite the fact that many of these chores also bore us). The overstimulation we subject our brains to prevents the necessary rest so that this creativity flows. The solution: boredom, because everyone knows that *boredom helps rest the brain*, as many pseudo-health blogs claim on the internet. But it is quite the opposite. The need to slam the door on boredom even manifests itself in states of stress and anxiety when we can't find the key to banishing it (Ros Velasco, 2022a). I also wonder why we were going to have to slow the pace for the light to come on. Pablo Picasso said that muses visit us while we work. Why not while we tweet or watch a video on TikTok?

I have not spoken about those who experience boredom in a dysfunctional fashion. We are not always in a position to respond to boredom, whether for better or for worse. Some people suffer from “individual-dependent chronic boredom” because they are unable to recognize more desirable situations than those that cause their boredom to encourage change (Ros Velasco, 2022a). Others, in turn, are able to identify the way in which they wish to break away from the source of boredom, but the very context that generates it prevent them from realizing the idea or putting it into practice, such that this boredom prevails indefinitely over time (this is what I have called “situation-dependent chronic boredom”, Ros Velasco, 2022a). How is the boredom of these situations going to make us more creative or is going to help rest the brain?

The last myths that I wish to broach are precisely related to the forms of dysfunctional boredom. For a long time, we have heard people say that *only those who want to are bored* in this life or, what is worse, that *only fools get bored*, those people who are empty inside, those who are unable to appreciate the immensity of the world and to wonder at the marvels of the divine creation, people who do not know how to manage their time optimally, delighting in the achievements of mankind. If only escaping from boredom were a simple question of willpower. For those who struggle to escape from boredom, these terrible myths are stigmatizing, aside from leading to a nauseating moral and intellectual superiority from those who perpetuate them. By means of these myths, the whole burden of responsibility in combatting boredom is placed on the subject, ignoring the importance of the context and that pathological states of boredom exist. The lament that *boredom is a privilege of the idle classes* also conveys an expression of moral superiority, albeit in a different sense. This is uttered by those who work all the time, convinced that only those who have nothing to do get bored. Those who proclaim this type of catchphrase blame others for not being productive, as if the exercise of productivity were a guarantee of a meaningful job. They not only err in their opinion, but are also guilty of envy.

These myths, which I classify as stigmatizing, are precisely those that halt the advance of Boredom Studies and of society itself. They make us ashamed of the causes of boredom, of things that do not satisfy us, and prevent us to discuss them as a group for fear of being pointed to as fools, vulgar, incapable or lazy. When someone asks us about what bores us, we hide our feelings and respond with such expressions as “I never get bored” or “I don't have time to be bored”, to show others that we hunger for knowledge and have very busy lives because we comply with the role we need to fulfill as useful cogs in the wheel of production. I am sure that there are real cases, worthy of study, of people who have no filter, able to engage with everything around them, but I doubt this is universal.

Sharing with others what we know about boredom, thanks to our own experiences, remarking on what bores us, destigmatizing boredom, and debunking the myths is the first step in preventing stagnation, dead-ends, situations which, as a society, we constantly create and turn into a source of boredom. This is a complicated exercise, as I said at the beginning, but essential for the continuity of progress. Throughout history, we have fallen into the trap of understanding boredom as a shameful, sinful, miserable and even sick state (Ros Velasco, 2022a), instead of as a useful experience to acknowledge the obsolescence of our own daily routines and our social constructs. The myth I will end this article with is the one that says that *we must learn to tolerate boredom*, one that gained fame since the COVID-19 pandemic began. This does not mean that we must be willing to suffer it daily or welcome it with open arms under the pretext that it will make us more creative, for example, but rather integrate its inevitable experience in public dialogue, shrugging off the prejudices and myths that prevent us from apprehending and harnessing it in its multifaceted fullness. They are the ones who lead us to try to escape boredom immediately, instead of accepting and exploring it closely to reap its benefits. Contemporary scholars of boredom dedicate ourselves to this commendable task of vindication.

## Data availability statement

The original contributions presented in the study are included in the article/supplementary material, further inquiries can be directed to the corresponding author.

## Author contributions

JRV contributed to conception, organization, writing, revision and translation, and approved the submitted version.
